# Delayed administration of recombinant plasma gelsolin improves survival in a murine model of severe influenza

**DOI:** 10.12688/f1000research.21082.2

**Published:** 2020-02-21

**Authors:** Zhiping Yang, Alice Bedugnis, Susan Levinson, Mark DiNubile, Thomas Stossel, Quan Lu, Lester Kobzik

**Affiliations:** 1 Department of Environmental Health, Harvard T.H. Chan School of Public Health, Boston, MA, 02115, USA; 2 BioAegis Therapeutics, North Brunswick, NJ, 07960, USA

**Keywords:** influenza, pneumonia, plasma gelsolin, immunomodulation, host-directed

## Abstract

**Background:** Host-derived inflammatory responses contribute to the morbidity and mortality of severe influenza, suggesting that immunomodulatory therapy may improve outcomes. The normally circulating protein, human plasma gelsolin, is available in recombinant form (rhu-pGSN) and has beneficial effects in a variety of pre-clinical models of inflammation and injury.

**Methods:** We evaluated delayed therapy with subcutaneous rhu-pGSN initiated 3 to 6 days after intra-nasal viral challenge in a mouse model of influenza A/PR/8/34.

**Results:** Rhu-pGSN administered starting on day 3 or day 6 increased survival (12-day survival: 62 % vs 39 %, pGSN vs vehicle; p < 0.00001, summary of 18 trials), reduced morbidity, and decreased pro-inflammatory gene expression.

**Conclusions:** Rhu-pGSN improves outcomes in a highly lethal influenza model when given after a clinically relevant delay.

## Introduction

Seasonal influenza continues to be a cause of substantial morbidity and mortality. There is also a fear that a new virulent influenza strain could cause high death rates, similar to those seen during the 1918 pandemic ^1^. The 2009 pandemic revealed the limitations of available public health interventions and current vaccines ^2^. While some antiviral drugs (e.g., oseltamivir) are currently in use, they suffer from a short time window of efficacy and increasing viral resistance ^3^. Hence, a substantial but unmet need exists for new therapeutic agents, especially for life-threatening infections.

The pathogenesis of influenza involves dysregulated and injurious host inflammatory responses ^4– 6^. This observation suggests that better inflammation control with immunomodulatory therapy may be able to reduce the morbidity and mortality seen in severe infections. Recombinant human plasma gelsolin (rhu-pGSN) is an attractive candidate because it dampens excessive and injurious inflammation and augments antimicrobial defenses. Moreover, it has successfully passed several of the safety, toxicity, and regulatory tests needed to go from ‘bench to bedside’.

Gelsolin was first identified in the cytoplasm of macrophages. It was further identified in many vertebrate cells, and is a highly conserved protein with many functions ^7, 8^. A unique characterstic of gelsolin at the gene level is the existence of a splice variant which encodes a distinct plasma isoform (pGSN). This isoform is released into extracellular fluids and differs from its cytoplasmic counterpart by the inclusion of an additional 25 amino acids at the N-terminal sequence. Normal mammalian blood contains pGSN at concentrations of 200–300 µg/ml, making it one of the most abundant proteins in plasma.

One of pGSN’s many functions is to dissolve the actin gels that arise from cellular debris, hence its name. These gels form a biofilm that reduces the ability of cellular and humoral defenses to gain access to embedded pathogenic organisms. In response, pGSN accumulates at sites of tissue damage. Interaction with actin reduces pGSN’s binding to and inactivation of a host of microbial toxins and inflammatory mediators (for example, lysophosphatidic acid, sphingosine-1-phosphate, platelet-activating factor, fibronectin, endotoxin and lipoteichoic acid). The local dynamic balance of these mediators can modulate host defense ^9, 10^. A complementary function of pGSN is its ability to augment the phagocytosis and killing of both Gram-positive and -negative bacteria by macrophages ^11^. By stripping actin off macrophage scavenger receptors, pGSN promotes phagocytosis. It also enhances killing by stimulating the constitutive NOS3 enzyme system ^11, 12^. As the acute injury subsides, pGSN is free to bind and inhibit inflammatory substances, promoting resolution of injury at the infectious site. The local capture of pGSN by exposed actin reduces the levels of pGSN in the circulation commensurate to the magnitude of tissue injury. The relative abundance ofpGSN typically allows it to render inactive any pro-inflammatory mediators that enter the systemic circulation and helps to prevent organ damage distant from the injury site. In severe infection, systemic depletion of pGSN can result in loss of its protective effects. Indeed, there is a robust correlation between how much pGSN levels decrease and probability of mortality. As might be predicted from these observations, systemic treatment withpGSN has reduced pathologic changes and mortality in numerous preclinical animal disease models ^7, 13, 14^.

Relevant to the severe pneumonia seen in fatal influenza, administration of rhu-pGSN improved survival in murine primary or secondary (post-influenza) pneumococcal pneumonia, a benefit seen without any antibiotic treatment ^11, 15^. These results have established proof-of-principle for the potential benefit of rhu-pGSN for bacterial pneumonias, including the secondary pneumonias often found as a complication of influenza. Here we report that rhu-pGSN improves outcomes in a mouse primary influenza model without superimposed bacterial infections.

## Methods

All protocols were approved by the Harvard Medical Area Biosafety and Animal Care and Use Committees.

### Mouse model of influenza

Normal 6- to 8-week-old male CD1 mice were obtained from Charles River Laboratories (Wilmington, MA). Only male mice were used due to budgetary and time limits. All mice arrived and were co-housed 1 week prior to the start of the experiments. Each trial used a separate batch of mice. A murine-adapted strain of H1N1 influenza virus, A/Puerto Rico/8/1934 (PR8), quantified as plaque-forming units (PFU) was procured from ViraSource (Durham, NC). Mice were anesthetized with 72 mg/kg ketamine plus 9.6 mg/kg xylazine administered via intraperitoneal injection. Mice then received an intranasal instillation of 25 μl suspension of PBS containing virus (ranging from 400–1000 PFU depending on the trial) or vehicle alone. All infections were done at approximately the same time of day (starting at ~10 AM). Initial titration identified 400 PFU as a dose that led to ~60% mortality in vehicle-treated mice, and this dose was used in a majority of the trials (see Table 1). Most trials used at least 10 mice per group for the vehicle and pGSN treatment groups; details of the influenza dose, total number of mice, and their weights are provided in the tables in *Underlying Data* ^16^.

**Table 1.** Details of treatment trials using recombinant human plasma gelsolin (rhu-pGSN) in murine influenza.

Trial #	Virus dose (PFU)	Treatment (start day)	pGSN dose (mg)	Treatment days	mice per group, n		Survival, %		Benefit *
					Vehicle	pGSN	Vehicle	pGSN	
1	400	6	5	d6-11	10	10	10	60	YES
2	400	6	5	d6-11	10	9	40	44	NO
3	500	3	5	d3-11	10	10	50	20	NO
	500	6	5	d6-11		10		44	NO
4	500	6	5	d6-11	10	10	20	40	YES
5	1000	6	5	d6-11	10	10	20	0	NO
6	400	6	5	d6-11	10	10	50	70	YES
7	600	6	5	d6-11	10	10	40	30	NO
8	400	6	5	d6-11	14	13	50	62	YES
	400	6	8	d6-11		10		80	YES
9	400	6	8	d6-11	15	15	40	67	YES
	400	6	10	d6-8		10		70	YES
10	400	6	5	d6-11	15	10	57	70	YES
	400	6	8	d6-11		15		80	YES
11	400	3	5	d3-11	10	10	60	90	YES
	400	3	8	d3-11		10		80	YES
	400	6	8	d6-11		10		70	YES
12	500	3	5	d3-11	10	10	40	90	YES
	500	3	5	d3-5		10		70	YES
	500	6	5	d6-11		10		60	YES
13	500	3	5	d3-11	22	19	59	50	NO
14	500	3	5	d3-11	18	7	39	57	YES
	500		5	d3-11		9		56	YES
	500		5	d3-11		8		38	NO
	500		5	d3-11		8		50	YES
15	400	3	2.5, 5	2.5 d3-5, 5 d6-11	19	13	42	77	YES
	400	6	5	5 d6-11		12	42	50	NO
16	400	3	2.5	2.5 d3-11	13	12	46	75	YES
	400	3	2.5, 5	2.5 d3-5, 5 d6-11		12	46	50	NO
	400	3	2.5, 5	2.5 d3-5, 5 d7-11		12	46	83	YES
17	400	3	0.5,3	0.5 d3-6, 3 d7-11	18	15	50	60	YES
	400	3	1,3	1 d3-6, 3 d7-11		15		47	NO
	400	3	2,5	2 d3-6, 5 d7-11		12		83	YES
18	400	3	1,5	1 d3-6, 5 d6-11	17	13	47	54	NO
	400	3	2,5	2 d3-6, 5 d7-11		10		70	YES

* Treatment benefit scored as Yes if % survival ≥10% better with pGSN vs. Vehicle; No if % survival <10% better with pGSN.

### Treatments and outcomes

Recombinant human pGSN (rhu-pGSN) was synthesized in *E. coli* and purified by Fujifilm Biosynth (Billingham, UK). We used human rather than murine gelsolin based on prior demonstrations of function of rhu-pGSN in rodent models and because data with the human gelsolin will facilitate clinical translation efforts. Rhu-pGSN was administered daily to mice by subcutaneous injection starting on day 3 or 6 after infection, at doses ranging from 0.5–5 mg as detailed in the *Results*. We monitored the mice for 12 days, measuring survival, changes in weight and overall morbidity using a composite index (i.e., 1 point each for hunched appearance, ruffled fur or partly closed eyes; 1.5 points for prolapsed penis or splayed hind quarter; 2 points for listlessness, with a maximum score of 8; the assessment was performed without blinding to treatment group) adapted from guidelines described previously ^17^. Weights and morbidity scores for the last day alive were carried forward for animals that did not survive.

### Lung transcriptome profiling

Lung tissue was obtained on days 7 and 9 after infection from mice treated with either vehicle or rhu-pGSN (dosed 2 mg per day starting on day 3 after infection, then increased to 5 mg per day on day 7). RNA was isolated using the RNAEasy mini-kit (Qiagen, Germantown, MD) according to manufacturer’s instructions. RNA samples were analyzed using the Mouse DriverMap targeted gene expression profiling panel from Cellecta (Mountain View, CA). The Cellecta platform uses highly multiplexed RT-PCR amplification and next-generation sequencing (NGS) quantitation to measure expression of 4753 protein-coding and functionally significant mouse genes. The procedure detailed in the Cellecta User Manual, item 5.3 was followed to create amplified index libraries which were sequenced on a Illumina NextSeq 500 instrument. The sequencing data was converted to FASTQ format and then further analyzed using DriverMap Sample Extraction software. This produces a raw data matrix file of counts for each sample in columns aligned to the 4753 gene panel.

### Statistical analysis

Data were analyzed using Prism (GraphPad Software) or SAS (SAS Institute) software. Differences in Kaplan-Meier survival curves were analyzed using a log-rank test with Sidak adjustment for multiple comparisons. A Breslow-Day test for homogeneity of the pGSN versus vehicle comparison across studies yielded p>0.2, indicating homogeneity could not be rejected and supporting the overall comparison across studies, which was carried out via the log-rank (Mantel-Cox) test stratified by trial. For other measurements, differences between groups were examined by ANOVA. The transcriptome profiling results scaled to normalize column counts, were converted to log2 counts (after addition of 0.1 to all cells to eliminate zero values) and then analyzed using Qlucore software (Lund, Sweden). Further analysis of gene set enrichment was performed using tools ( Panther version 14.1 ^18^ and MetaCore (version 19.3, Clarivate Analytics, Philadelphia, PA)) that allow evaluation using a custom background gene list (i.e., the ~4700 genes measured using the Cellecta DriverMap platform).

## Results

### Effect of rhu-pGSN on survival

We tested a variety of dose and timing regimens to evaluate the potential of rhu-pGSN to improve outcomes, conducting a total of 18 trials that are tabulated in Table 1 and summarized in Table 2. To mimic likely clinical usage, mice were not treated until several days post-challenge

**Table 2.** Summary of survival data using different treatment regimens.

Subset analyzed	Experiments analyzed, n	Viral dose, PFU(cohorts tested, n)	Vehicle start, n	Vehicle survived, n	Vehicle survival, %	pGSN start, n	pGSN survived, n	pGSN survival, %	p-value
**All data**	**18**	**400 (21); 500 (11);** **600 (1); 1000 (1)**	**236**	**93**	**39**	**389**	**241**	**62**	**<0.000001**
**Treatment d6-11**	**11**	**400 (11); 500 (4);** **600 (1); 1000 (1)**	**148**	**59**	**40**	**172**	**110**	**64**	**0.000011**
**Treatment d3-11 2mg+**	**9**	**400 (7); 500 (7)**	**137**	**58**	**42**	**162**	**101**	**62**	**0.0005**
**Treatment start low 2** **mg+ (d3-6/7) then high** **dose**	**4**	**400 (6)**	**67**	**34**	**51**	**71**	**55**	**77**	**0.0005**
**Treatment start very** **low (0.5-1) mg+ (d3-6/7)** **then high dose**	**3**	**400 (3)**	**35**	**19**	**54**	**43**	**24**	**56**	**0.62**

pGSN, plasma gelsolin

**Figure 1.**
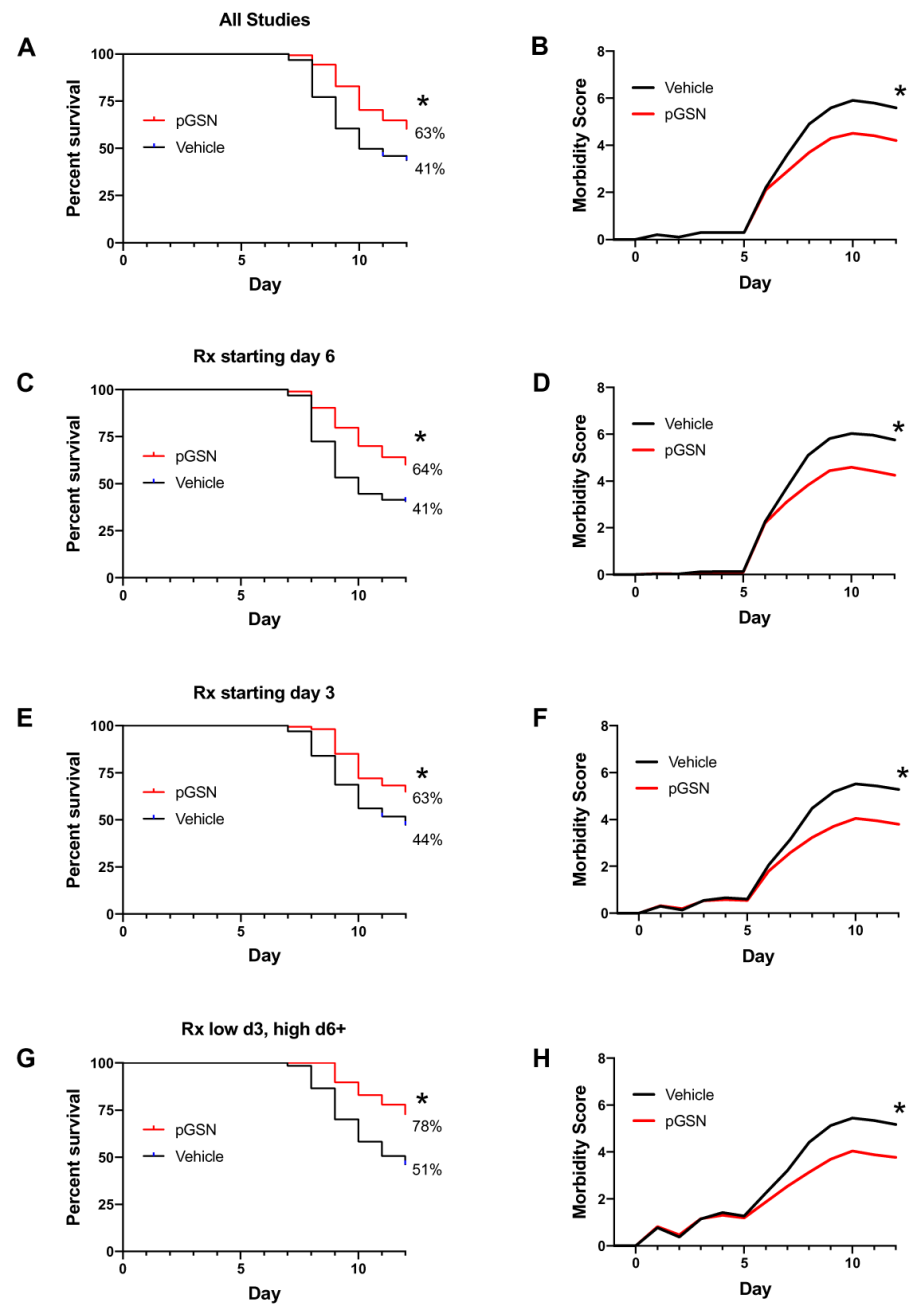
Survival and morbidity analysis of different treatment regimens. Comparison of survival rates ( **A**, **C**, **E**, **G**) and morbidity ( **B**, **D**, **F**, **H**) in mice treated with rhu-pGSN or vehicle. ( **A**, **B**) Results for all 18 trials (typically 10 or more mice per group, see details in Table 1 and Table 2) using delayed treatment. Some trials initiated treatment in different arms on day 6 or day 3. ( **C**, **D**) Results for 13 trials using delayed treatment starting on day 6 or later. ( **E**, **F**) Results for eight trials using treatment starting on day 3. ( **G**, **H**) Results for four trials starting with an initially lower dose on day 3 with an increased dose starting on day 6/7. * = 0.000001, 0.00001, 0.0005, 0.0005 for A, C, E, G, respectively; p < 0.0001 for B, D, F, H.

The main finding was that delayed treatment with rhu-pGSN resulted in significant improvement in the survival of mice ( Figure 1). All studies combined yielded 39% (93/236) surviving mice treated with vehicle and 62% (241/389) surviving mice treated with pGSN on day 12 (p = 0.000001, Figure 1A). Improved survival was observed whether the delayed treatment was started on day 6 ( Figure 1C) or day 3 after infection ( Figures 1E, G). Similarly, compared to vehicle treatment, rhu-pGSN resulted in decreased morbidity scores ( Figures 1B, D, F, H). In contrast, no statistically significant difference in weight loss or recovery (in surviving animals) was consistently observed in the experiments summarized in Figure 1. The sole exception was found in the trials testing a dose regimen of initially low (> 2 mg rhu-pGSN on days 3–6/7, then 5 mg through day 11). The latter set of trials led to weights (compared to day 0) at the end of study of 81.4 ± 4.7% in vehicle-treated mice versus 85 ± 2.6% in pGSN-treated mice (p < 0.0001, summary of 4 trials, see also Table 1 and Table 2, and more detailed tabulation of all experiments in *Extended data* ^16^. A beneficial effect of rhu-pGSN was observed in a majority but not all of the 18 individual trials ( Table 1, see *Discussion*).

### Transcriptome profiling

**Figure 2.**
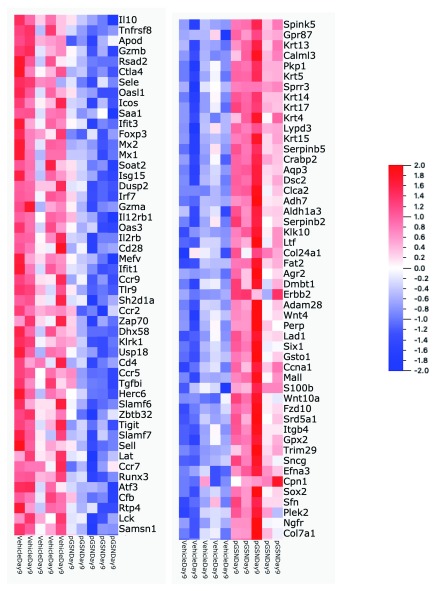
Top 50 up- and down- regulated differentially expressed genes in lung tissue from vehicle or rhu-pGSN treated animals (Day 9). Heat map showing top 50 down-regulated (left) and up-regulated (right) genes in the lungs of rhu-pGSN treated animals on day 9 (range -2 (blue) to + 2 (red)).

To evaluate whether rhu-pGSN treatment modified the transcriptome profile (see *Underlying data*) of infected lungs, we harvested lung tissue just before (day 7) and after (day 9) the usual onset of mortality (day 8) in this model (n = 5 per group per day). Per protocol, the rhu-pGSN dose was increased in this experiment on day 7, between the 2 timepoints selected for profiling. Comparison of lung samples obtained at day 7 from vehicle-treated and rhu-pGSN-treated mice showed no significant differences. In contrast, analysis of day 9 samples identified 344 differentially expressed genes in the rhu-pGSN-treated group, comprised of 195 down-regulated and 149 up-regulated genes. The top 50 up- and down-regulated genes are shown in Figure 2, which is notable for the many cytokine and immune-related genes prominent among those down-regulated in the rhu-pGSN-treated group (including IL10, IL12rb, CTLA4, and CCRs9, 7 and 5, among others). We performed gene enrichment analysis of the full down-regulated gene list using the Panther online analysis tool to query GO Ontology or Reactome databases. The main findings were a reduction of expression of biological processes linked to immune and inflammatory responses, or release of cytokine and other cellular activators. The top 10 most significant processes/pathways are shown in Table 3. Analysis using a different gene enrichment analysis software tool (MetaCore) produced similar results. Analysis of the up-regulated gene list identified enrichment of processes related to tissue morphogenesis and epithelial/epidermal cell differentiation (consistent with repair of influenza-mediated damage, see *Discussion*). We present details of the DriverMap gene list, the differentially expressed genes identified, and the full results of gene enrichment analyses using the down- and up-regulated gene lists to query the Panther and MetaCore databases in worksheets 2–15 in a spreadsheet available in *Extended data* ^19^.

**Table 3.** Top 10 down-regulated Gene Ontology (GO) processes and pathways in plasma gelsolin (pGSN)-treated lung tissue (Day 9).

		Day 9 downregulated genes in pGSN-treated group				
GO biological process	Process genes in background list, n	Genes in day 9 downreg genes in pGSN group, n	Expected genes, n	Fold enrichment	Raw P-value	FDR
immune system process (GO:0002376)	932	126	38.5	3.27	4.13E-40	5.59E-36
immune response (GO:0006955)	496	88	20.49	4.29	9.01E-33	6.09E-29
defense response (GO:0006952)	534	83	22.06	3.76	6.69E-27	3.02E-23
response to external biotic stimulus (GO:0043207)	500	78	20.66	3.78	4.19E-25	9.44E-22
response to biotic stimulus (GO:0009607)	511	79	21.11	3.74	3.03E-25	1.03E-21
response to other organism (GO:0051707)	500	78	20.66	3.78	4.19E-25	1.13E-21
regulation of immune system process (GO:0002682)	682	90	28.18	3.19	8.49E-25	1.64E-21
positive regulation of immune system process (GO:0002684)	485	75	20.04	3.74	8.83E-24	1.49E-20
response to external stimulus (GO:0009605)	928	100	38.34	2.61	8.64E-22	1.30E-18
defense response to other organism (GO:0098542)	343	61	14.17	4.3	1.14E-21	1.54E-18
**Reactome pathways**						
Immune System (R-MMU-168256)	773	85	31.94	2.66	2.92E-18	4.52E-15
Cytokine Signaling in Immune system (R-MMU- 1280215)	257	38	10.62	3.58	3.48E-11	2.69E-08
Adaptive Immune System (R-MMU-1280218)	299	37	12.35	3	6.13E-09	3.16E-06
Metabolism (R-MMU-1430728)	783	8	32.35	0.25	1.48E-07	5.72E-05
Immunoregulatory interactions between a Lymphoid and a non-Lymphoid cell (R-MMU-198933)	55	14	2.27	6.16	3.39E-07	1.05E-04
Innate Immune System (R-MMU-168249)	453	43	18.72	2.3	5.72E-07	1.48E-04
Signaling by Interleukins (R-MMU-449147)	192	24	7.93	3.03	3.33E-06	7.37E-04
GPVI-mediated activation cascade (R-MMU-114604)	26	9	1.07	8.38	5.84E-06	1.13E-03
DAP12 interactions (R-MMU-2172127)	20	7	0.83	8.47	6.27E-05	1.08E-02
Interleukin-2 family signaling (R-MMU-451927)	30	8	1.24	6.45	9.32E-05	1.31E-02

## Discussion

We sought to evaluate the potential of rhu-pGSN to improve outcomes in severe influenza using a clinically relevant scenario of delaying initiation of treatment. The key finding was that delayed pGSN treatment significantly improved survival, either when used starting on day 3 or even starting as late as day 6 after infection. In addition to the impractically of initiating earlier therapy right after infection (as opposed to the onset of severe symptoms) in patients, we did not want to interfere with the immediate immune response to influenza given the detrimental consequences observed in some experimental models.

Some limitations merit discussion. The first is the experimental variability we observed and report. Treatment with rhu-pGSN increased survival in a majority of the experiments conducted, but not in all of them. For a subset of the negative trials, we could postulate plausible potential explanations (e.g., technical issues with the virus stock, variation in instillation method, insufficient initial rhu-pGSN dose in the ‘low dose then high dose’ trials). To the extent possible, we adjusted our methods to reduce these potential sources of variability. However, for the remainder of the negative trials, we simply do not have a good explanation for the outcome. Hence, we have chosen to present all the data whether positive or negative to provide a full report of the findings.

We also manipulated the experimental variables, in part to address larger questions (e.g., can treatment as late as day 6 vs day 3 after onset of infection be effective?) and in some cases to explore potential reasons for the intermittent variability in our results (e.g., trial 14 tested the potential influence of differences in initial weight of the mice we used). Ultimately, we observed beneficial effects whether the survival analysis included all the trials ( Figures 1A, B) or those using treatment starting at day 6 or day 3 ( Figures 1C–H).

Mice were only followed for 12 days when euthanasia was performed on surviving mice. Since the survival curves were still potentially declining, the ultimate mortality rate could not be confidently ascertained. However, the time to death at a minimum was prolonged with rhu-pGSN over placebo treatment.

Notably, rhu-pGSN did not rescue all of the mice dying from influenza in our model, offering only a partial (albeit significant) survival benefit. Given the goal of identifying a novel therapy for severe influenza, an optimistic interpretation is that this occurred in mice without the supportive fluid and respiratory care given to hospitalized patients, and that similar or more robust benefits might be observed in the clinical setting. We can also speculate that combination therapy might offer a greater survival advantage. The results establish a potential benefit for rhu-pGSN but this potential needs further evaluation in a larger animal model, e.g. ferret ^20^ and then (if results warrant), testing in a clinical trial to determine its role in therapy for severe influenza in human patients. Our findings rely on studies with only one strain of influenza in only one strain of one model species, the mouse. Nevertheless, we favor future experimentation in a larger animal model as the logical next step, rather than further studies in mice. Additional investigations using other influenza or mouse strains would not resolve the suggestion (hope) of possible clinical benefit offered by our results. Hence, large animal experiments deserve priority.

Our study did not address the mechanism(s) for the beneficial action of rhu-pGSN. The available literature identifies numerous inflammatory mediators whose function can be modulated by pGSN (e.g. sphingosine-1-phophate ^9^, endotoxin ^10^, platelet activating factor ^21^). The transcriptome profiling results are consistent with a beneficial down-regulation of the overly exuberant immune and inflammatory response that characterizes severe influenza ^22– 24^. Further investigation of the many possible single or combination targets by which pGSN may be acting is warranted. However, a complete delineation of its mechanisms will take substantial effort and time to achieve. If effective, therapeutic use of rhu-pGSN should be pursued even in the absence of a full map of its complex effects. This position reflects in part the fact that pGSN is a normal, abundant protein in human plasma, and has passed initial safety evaluation in human subjects hospitalized for non-severe community acquired pneumonia (ClinicalTrials.gov NCT03466073). Finally, it is worth speculating that rhu-pGSN treatment may also benefit patients with severe influenza by reducing the risk of the common complication of secondary bacterial pneumonia ^25, 26^. This possibility is suggested by other studies from our laboratory, showing rhu-pGSN improved survival of mice with post-influenza bacterial pneumonia ^11^.

In summary, rhu-pGSN can improve outcomes in a highly lethal murine influenza model when given after a clinically relevant delay. These findings are consistent with the benefits seen in models of pneumococcal pneumonia. The modes of action for pGSN involve host responses and do not seem to depend on the specific type of pathogen. Our findings support further investigation of pGSN as an adjunctive therapy for severe influenza and other viral infections.

## Data availability

### Underlying data

Harvard Dataverse: Expanded Tables 1 & 2. https://doi.org/10.7910/DVN/53GJY1 ^16^.

This project contains data on each experimental group, as shown in Table 1 and Table 2, with additional variables, such as weight, and statistical analyses.

NCBI Gene Expression Omnibus: Transcriptome profiling of lung tissue from influenza-infected mice treated with plasma gelsolin. Accession number GSE138986; https://identifiers.org/geo:GSE138986.

### Extended data

Harvard Dataverse: Transcriptome analysis of gelsolin vs vehicle treatment in mouse influenza infected lungs. https://doi.org/10.7910/DVN/8HBFD7 ^19^.

### Reporting guidelines

Harvard Dataverse: ARRIVE checklist for ‘Delayed administration of recombinant plasma gelsolin improves survival in a murine model of severe influenza’. https://doi.org/10.7910/DVN/VQBKLF ^27^.

Data hosted on Harvard Dataverse are available under the terms of the Creative Commons Zero "No rights reserved" data waiver (CC0 1.0 Public domain dedication).
