# Supplementary material for: Delayed administration of recombinant plasma gelsolin improves survival in a murine model of severe influenza
Source: F1000Res. 2020 Feb 21;8:1860. Originally published 2019 Nov 6. [Version 2] doi: 10.12688/f1000research.21082.2 (PMC6894358; doi:10.12688/f1000research.21082.2)
Supplement: Peer review history

# Peer review: Reviewer response for version 2

**DOI:** 10.5256/f1000research.24817.r60385

**Published:** 2020-02-21

**Authors:** David H. Dockrell

**Affiliations:** 1 MRC Centre for Inflammation Research, University of Edinburgh, Edinburgh, UK

The authors appear to have addressed the issues raised and clarified the main points raised in review.

I confirm that I have read this submission and believe that I have an appropriate level of expertise to confirm that it is of an acceptable scientific standard.

# Peer review: Reviewer response for version 1

**DOI:** 10.5256/f1000research.23201.r56253

**Published:** 2019-12-04

**Authors:** David Fedson

**Affiliations:** 1 None (retired, prolific writer on influenza and host-directed therapies), Sergy Haut, France

**F1000RESEARCH – Submitted by David S. Fedson, MND**

 Yang Z, Bedugnis A, Levinson S, DiNubile M, Stossel T, Li Q, Kobzik L. **Delayed administration of recombinant plasma gelsolin improves survival in a murine model of severe influenza.** *F1000 Research* 2019; **8:**1860.

**Reviewer’s comments**

 This straightforward paper presents the results of a study showing that rhu-pGSN improves survival in a mouse model of influenza. My answers to the mandatory questions above indicate the paper is acceptable and requires no significant modification. The comments below are *suggestions *and are offered in the hope they might help readers. The authors should feel free to ignore them if they wish. 

**Introduction, page 3, left column, paragraph 2 - **The pathogenesis of influenza involves a dysregulated and injurious host response that is not wholly inflammatory. Inflammation is involved to be sure, but there is also a degree of immunosuppression. Some of the changes (e.g., pulmonary endothelial dysfunction) can be regarded as separate from inflammation and/or immunosuppression. The authors argue that rhu-pGSN is “an attractive candidate (for treatment) because it dampens excessive and injurious inflammation and augments antimicrobial defences”. Rhu-pGSN probably does much more. (See Becker PM *et al*. Pulmonary vascular permeability and ischemic injury in gelsolin-deficient mice. Am J Respir Cell Mol Biol 2005;28:478-84 ^1^ and earlier Kuhne W *et al.* Disintegration of cytoskeletal structure of actin filaments in energy-depleted endothelial cells. Am J Physiol 1993;265(5 Pt2):H1599-608 ^2^.)

**Page 3, left column, paragraph 4, lines 1-7 - **The first 7 lines could be written more tightly. “One of pGSN’s many functions is to dissolve the actin gels that arise from cellular debris, hence its name. These gels form a biofilm that reduces the ability of cellular and humoral defences to gain access to embedded pathogenic organisms. In response, pGSN accumulates at sites of tissue damage. Interaction with actin reduces pGSN’s binding to …” Also, in line 12, delete “final”. This is not the final comment in this paragraph. 

**Page 3, right column, paragraph 1, lines 3-4** - A sentence could be added: “… levels decrease and probability of mortality. For this reason, pGSN levels have been considered as potential biomarkers of severity for several acute and chronic diseases, ^7^ In addition, as might be predicted, systemic treatment …” (Reference 7 is a remarkably complete review of published studies of gelsolin and should be read by anyone reading this paper.)

**Page 3, right column, paragraph 2, lines 1-2** - These lines could be rewritten: “Regarding the treatment of the severe pneumonia often seen in fatal influenza, administration of rhu-pGSN …”

**Results, page 5, right column, paragraph 2, lines 12-15 **– These lines could be rewritten: “…The only exception was found in trials 15-19 (Table 1) that tested dose regimens that were initially low (>2 mg rhu-pGSN on days 3 to 5/6, then 5 mg daily through day 11). These trials led to weights (compared to day 0) at the …”  By including “(compared to day 0)”, it is not entirely clear whether the weights mentioned in lines 16 and 17 (81.4 and 85 g?) are mean weight differences between day 0 and the end of the study in each of the two groups or the mean weight difference between the two groups at the end of the study. I assume it is the former, but the reader would have to check *Extended data* to be certain. 

**Page 5, paragraph 2, line 19 and paragraph 3, line 2 - **What is the difference (if any) between *Extended data* and *Underlying data*? Should *Underlying data* really be *Extended data*?

**Page 5, paragraph 3, line 5 – **This sentence could be rewritten: **“… **Comparison of lung samples obtained on day 7 from vehicle-treated and rhu-pGSN-treated mice showed …”

**“Page 5, paragraph 3, lines 10-1 – **This sentence could be rewritten: “… Among down-regulated genes in the rhu-pGSN-treated group, many cytokine and immune-related genes were prominent, including IL-10, IL-12b, …” 

**Page 7, left column paragraph 1, line 3 – **Do the “main findings” in the analysis of down-regulated genes refer to all down-regulated genes or only the top 50? I assume it is the latter, but this should be made clear to the reader.

**Page 7, left column, paragraph one, last four lines (and top two lines in the right column) – **This is a very long sentence. It’s worth considering a rewrite: “…In worksheets 2-15 in the spreadsheet in *Extended data*, we present details of the … “  

**Page 8, right column, paragraph 1, line 2 – **It should be “virus stock”, not “viral stock”.

 The authors should be commended for admitting they can’t explain all of their results. 

**Page 8, right column, paragraph 1-** The authors showed rhu-pGSN treatment offered “only a partial (albeit significant) survival benefit”. Among their suggestions for future studies they mention using larger animals and other strains of influenza virus. They overlook the possibility of combining rhu-pGSN with other drug treatments. After all, they showed only a modest increase in survival (roughly 40% in vehicle-treated and 60% in rhu-pGSN-treated mice, respectively). Combination treatment might offer a greater survival benefit. The authors might also emphasize that studies of the effects of gelsolin and treatment with rhu-pGSN on host responses to several infections suggest rhu-pGSN might used in the syndromic treatment of many different infectious diseases. 

**References**

 In addition to the two articles on gelsolin and endothelial dysfunction mentioned earlier, the authors might include their more recently published paper:

 Self WH, Wunderink RG, DiNubile MJ, Stossel TP, Levinson SL, Williams DJ, *et al.* **Low admission plasma gelsolin concentrations identify community-acquired pneumonia patients at high risk for severe outcomes.** *Clin Infect Dis*2019; **69**(7):1218-25 ^3^.  

**Tables and Figures**

**Table 1. **Dividing the table into two sections showing results of treatments started on day 3 and day 6 might make it easier for readers to see the important findings. The two sets of findings could be further subdivided into those showing benefit (YES) or not (NO). 

**Figure 1** - The legends within panels A, C, E, and G show pGSH above vehicle, while in panels B, D, F, and H the two legends are reversed. It would be better if a consistent style could be used throughout the figure. If the legends are changed, the authors should use rhu-pGSN, which is what they used in their studies (not pGSN).  

**Figure 2.** – It would be helpful to include headings (down-regulated and up-regulated) at the top of the two sets of heat maps.

I confirm that I have read this submission and believe that I have an appropriate level of expertise to confirm that it is of an acceptable scientific standard.

## References

[1] : Pulmonary vascular permeability and ischemic injury in gelsolin-deficient mice. *Am J Respir Cell Mol Biol*.2003;28(4) : 10.1165/rcmb.2002-0024OC 478-84 10.1165/rcmb.2002-0024OC 12654637

[2] : Disintegration of cytoskeletal structure of actin filaments in energy-depleted endothelial cells. *Am J Physiol*.1993;264(5 Pt 2) : 10.1152/ajpheart.1993.264.5.H1599 H1599-608 10.1152/ajpheart.1993.264.5.H1599 8388659

[3] : Low Admission Plasma Gelsolin Concentrations Identify Community-acquired Pneumonia Patients at High Risk for Severe Outcomes. *Clin Infect Dis*.2019;69(7) : 10.1093/cid/ciy1049 1218-1225 10.1093/cid/ciy1049 30561561PMC6743831

# Response

**Published:** 2020-02-17

**Authors:** Lester Kobzik

**Affiliations:** Environ. Health, Harvard University, USA

Response to Reviewer #2:

*This straightforward paper presents the results of a study showing that rhu-pGSN improves survival in a mouse model of influenza. My answers to the mandatory questions above indicate the paper is acceptable and requires no significant modification. The comments below are suggestions and are offered in the hope they might help readers. The authors should feel free to ignore them if they wish.*

We are grateful for the many constructive comments and suggestions. As allowed by the reviewer, we have left some comments regarding minor layout and sentence construction issues unanswered.  However, for the majority we undertook revisions in the manuscript that are reported below:

***Page 3, left column, paragraph 4, lines 1-7 -*** *The first 7 lines could be written more tightly. “One of pGSN’s many functions is to dissolve the actin gels that arise from cellular debris, hence its name. These gels form a biofilm that reduces the ability of cellular and humoral defences to gain access to embedded pathogenic organisms. In response, pGSN accumulates at sites of tissue damage. Interaction with actin reduces pGSN’s binding to …”*

This suggested text has been used in its entirety to replace the prior version.

***Page 5, paragraph 2, line 19 and paragraph 3, line 2 -*** *What is the difference (if any) between Extended data and Underlying data? Should Underlying data really be Extended data?*

These are rubrics used by F1000 and we followed editorial guidelines in designated data for one or the other grouping. The links to the data at the end of the paper allow the reader to see these data. We cannot better explain the rationale for ‘underlying vs extended”.

***Page 5, paragraph 3, line 5 –*** *This sentence could be rewritten: **“… **Comparison of lung samples obtained on day 7 from vehicle-treated and rhu-pGSN-treated mice showed …”*

This suggested text has been used in its entirety to replace the prior version.

***Page 7, left column paragraph 1, line 3 –*** *Do the “main findings” in the analysis of down-regulated genes refer to all down-regulated genes or only the top 50? I assume it is the latter, but this should be made clear to the reader*.

As suggested, we have clarified this point by changing the text as below to indicate that all the down-regulated genes were used for this analysis:

We performed gene enrichment analysis of the full down-regulated gene list

***Page 8, right column, paragraph 1, line 2 –*** *It should be “virus stock”, not “viral stock”.*

Fixed as suggested.

***Page 8, right column, paragraph 1-*** *The authors showed rhu-pGSN treatment offered “only a partial (albeit significant) survival benefit”. Among their suggestions for future studies they mention using larger animals and other strains of influenza virus. They overlook the possibility of combining rhu-pGSN with other drug treatments. After all, they showed only a modest increase in survival (roughly 40% in vehicle-treated and 60% in rhu-pGSN-treated mice, respectively). Combination treatment might offer a greater survival benefit.*

We have added the sentence below to this section to include this helpful idea.

We can also speculate that combination therapy with other agents might offer a greater survival advantage.  

Unrelated to any specific reviewer comment, we added more detailed mention early in the Discussion of why we did not test rhu-pGSN treatment before day 3:

In addition to the impractically of initiating earlier therapy right after infection (as opposed to the onset of severe symptoms) in patients, we did not want to interfere with the immediate immune response to influenza given the detrimental consequences observed in some experimental models.

# Peer review: Reviewer response for version 1

**DOI:** 10.5256/f1000research.23201.r56255

**Published:** 2019-11-18

**Authors:** David H. Dockrell

**Affiliations:** 1 MRC Centre for Inflammation Research, University of Edinburgh, Edinburgh, UK

Yang *et al.* explore the potential for of recombinant human gelsolin as a therapeutic intervention in a PR8 H1N1 mouse flu model. They use CD1 mice and use a variety of doses and dosing schedules with a pooled statistical analysis to infer benefits across a range of groups despite some variability in results. Benefits were seen in terms of survival and morbidity scores but not in terms of weight loss. They went on to perform transcriptional profiling and to detect reductions in inflammatory responses and increases in responses associated with tissue repair.

 Strengths of the study are the investigation of a therapeutic approach for a potential life threatening infection and the preliminary hypothesis generating transcriptomic data set which provide clues to potential mechanisms. A few areas of the text should be addressed to strengthen the main conclusions.

 The authors have had significant statistical input but some further explanation of the statistical approach and its potential benefits to a study such as this would be useful.

 The study used CD1 mice which may explain some of the variability. A few further details should be added to the methods. The authors should confirm the reason for just studying males. They should confirm animals and groups were co-housed and, in a study with such large numbers, explain whether there were any majors groupings or time periods used in the study which might have contributed to variability. Were mice bought in in small groups or are the subgroups from predominantly the same larger pool bred in house? Also they should confirm the mice were infected at similar times of day.

 The authors might also comment on the rationale for using human gelsolin. Some differences in response between human and mouse gelsolin have been detected and the authors might wish to comment on this and their similarities and differences.

 The authors did not find any major differences in weight. Can the authors comment further on this since this is usually a marker of outcome. In addition the mortality curves appeared to be still showing deaths at the end of the study period. Can the authors confirm that the main biologic affect was a delay in mortality rather than prevention? Clarifying this in the text would resolve any uncertainty relating to the conclusion and title.

 The transcriptomic data is of interest. Can the authors drill down further and confirm any sub-groupings related to inflammation or tissue repair? In particular is their any evidence of an epithelial protective affect such as enhancement of barrier function or epithelial apoptosis inhibition, since this is potentially one of gelsolins biologic functions. I assume there is no additional data from BAL cytokines, cell counts or histology to back up the transcriptomics but if there are any further data, they would strengthen the conclusions.

I confirm that I have read this submission and believe that I have an appropriate level of expertise to confirm that it is of an acceptable scientific standard.

# Response

**Published:** 2020-02-17

**Authors:** Lester Kobzik

**Affiliations:** Environ. Health, Harvard University, USA

Response to Reviewer #1:

*The study used CD1 mice which may explain some of the variability. A few further details should be added to the methods.*

*The authors should confirm the reason for just studying males.*

*They should confirm animals and groups were co-housed and, in a study with such large numbers, explain whether there were any majors groupings or time periods used in the study which might have contributed to variability. Were mice bought in in small groups or are the subgroups from predominantly the same larger pool bred in house?*

*Also they should confirm the mice were infected at similar times of day.*

To address these points, the following text has been inserted into the methods: 

Only male mice were used due to budgetary and time limits. All mice arrived and were co-housed 1 week prior to the start of the experiments. Each trial used a separate batch of mice.

All infections were done at approximately the same time of day (starting at ~10 AM).

*The authors might also comment on the rationale for using human gelsolin. Some differences in response between human and mouse gelsolin have been detected and the authors might wish to comment on this and their similarities and differences*.

To address this comment, the following text has been inserted into the methods: 

We used human rather than murine gelsolin based on prior demonstrations of function of rhu-pGSN in rodent models and because data with the human gelsolin will facilitate clinical translation efforts.

*The authors did not find any major differences in weight. Can the authors comment further on this since this is usually a marker of outcome. In addition the mortality curves appeared to be still showing deaths at the end of the study period. Can the authors confirm that the main biologic affect was a delay in mortality rather than prevention? Clarifying this in the text would resolve any* uncertainty relating to the conclusion and title.

We were also puzzled by the lack of weight differences but have no explanation to offer. A small number of the initial trials followed mice longer than the period reported in the paper. Mice surviving to day 12 continued to live up to day 18-21 in these early trials. However, this was not systematically studied so it is fair to say it is a qualitative impression rather than a quantitative result. Extending the trial length would require additional experimentation. Either a significant delay in mortality or true improved survival would provide the same basis for supporting further studies of rhu-pGSN as a therapeutic worth validating in other models (e.g. ferret).

Re: the reviewer’s point about mortality, we now explicitly acknowledge in the Discussion:

Mice were only followed for 12 days when euthanasia was performed on surviving mice.  Since the survival curves were still potentially declining, the ultimate mortality rate could not be confidently ascertained. However, the time to death at a minimum was prolonged with rhu-pGSN over placebo treatment.

*The transcriptomic data is of interest. Can the authors drill down further and confirm any sub-groupings related to inflammation or tissue repair? In particular is their any evidence of an epithelial protective affect such as enhancement of barrier function or epithelial apoptosis inhibition, since this is potentially one of gelsolins biologic functions. I assume there is no additional data from BAL cytokines, cell counts or histology to back up the transcriptomics but if there are any further data, they would strengthen the conclusions.*

We did not find any enrichment for the groupings suggested beyond what we reported in the supplemental data spreadsheet. No additional data from the samples listed by the reviewer is available are available, so this interesting topic can only be addressed in future studies. A comment on treatment changes between the two time points for transcriptome profiling was inserted in the Results section:

Per protocol, the rhu-pGSN dose was increased in this experiment on day 7, between the 2 timepoints selected for profiling.
